# Crusted Scabies Induced Hypereosinophilic Syndrome

**DOI:** 10.7759/cureus.15201

**Published:** 2021-05-23

**Authors:** Asis Shrestha, Edward Bischof

**Affiliations:** 1 Internal Medicine, Rochester Regional Health, Rochester, USA; 2 Internal Medicine, Bassett Medical Center, Cooperstown, USA

**Keywords:** crusted scabies, erythroderma, hypereosinophilic syndrome, hypereosinophilia, hyperkeratotic skin lesions

## Abstract

Crusted scabies causes extensive hyperkeratotic skin lesions, crusting, and scaling and is common in elderly and institutionalized patients. We present a case of crusted scabies in a patient with encephalopathy and diffuse exfoliative erythroderma. After extensive workup, the patient’s condition was attributed to hypereosinophilic syndrome due to crusted scabies. Skin condition, mental status, and eosinophilia improved with high-dose steroids in conjunction with topical permethrin and oral ivermectin.

## Introduction

Human scabies is a common ectoparasitic infestation by the mite Sarcoptes scabiei. Globally, it is estimated to affect more than 200 million people at any time, but has high variation in prevalence among individual geographic regions [1], and is mostly seen in residential and nursing care facilities for the elderly [2]. Classical scabies presents as pruritic eruptions with papules, nodules, and subcutaneous burrows. Crusted scabies is a less common variant that affects people who have reduced cellular immunity, and is characterized by thick crusty, scaly, and fissured skin.

Hypereosinophilia has been defined as absolute eosinophil count > 1500 cells/uL on two examinations separated in time by at least one month and/or pathologic confirmation of tissue hypereosinophilia. Hypereosinophilic syndrome (HES) is defined as the association of hypereosinophilia with eosinophil-mediated target organ damage or dysfunction [3]. Scabies, like other parasitic infections, can cause hypereosinophilia, but whether it can lead to HES is unknown. We present a case of a 73-year-old male with crusted scabies who presented with a constellation of symptoms that was attributed to HES. The goal of this report is to raise awareness of an easily overlooked disease, crusted scabies, that can have significant morbidity, including HES.

## Case presentation

A 73-year-old male with a past medical history of hypertension and chronic mild diastolic congestive heart failure presented with three consecutive admissions for altered mental status. He was initially admitted for methicillin-resistant Staphylococcus aureus (MRSA) bacteremia secondary to lower extremity cellulitis. Altered mental status was thought of due to metabolic encephalopathy from sepsis which improved with intravenous vancomycin and was then discharged to a rehabilitation center. He presented again a few weeks later with altered mental status, hypothermia, and hypoglycemia. He was initiated on intravenous hydration and steroid which improved his clinical condition. Adrenal insufficiency was ruled out and he was then discharged off steroids back to the rehabilitation facility. At the facility, he gradually developed worsening confusion, lethargy, and somnolence, with poor oral intake, which brought him to the hospital again for the third time. In addition to his above history, he also had chronic hyperkeratotic skin lesions with calluses and fissures for which he applied ammonium lactate lotion for two years. His home medications during the presentation included only furosemide 20 mg once a day.

**Figure 1:**
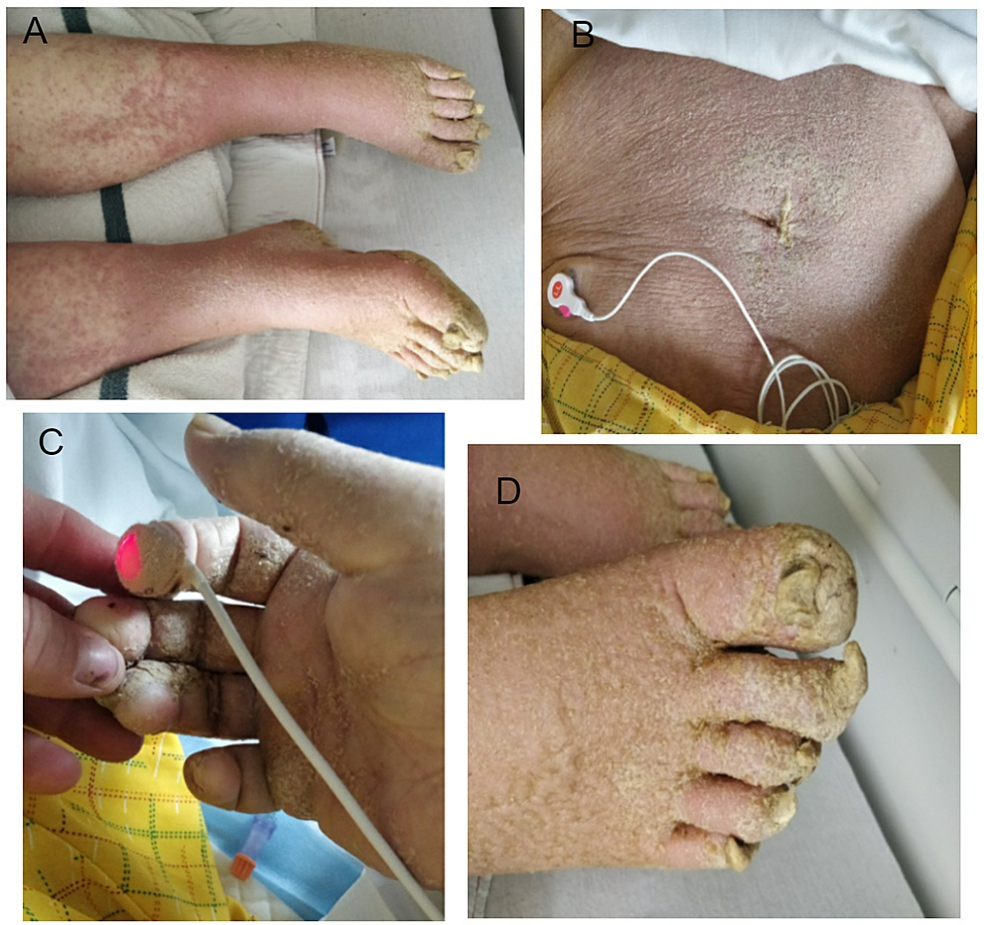
Erythroderma with hyperkeratotic crusty scaly lesions seen on bilateral lower legs (A), abdomen (B), hand (C) and feet (D).

On presentation during this third admission, he presented with normal blood pressure and pulse but hypothermia (34 °C) and decreased level of consciousness. He would open his eyes occasionally but did not follow commands. No focal neurologic deficits were identified. His skin examination revealed generalized erythroderma with hyperkeratotic, crusty, scaly lesions on his abdomen and all extremities including palms and soles (Figure 1).

Laboratory values were significant for acute kidney injury (creatinine 2.3 mg/dl, baseline 1.7 mg/dl), hypoglycemia (glucose 57 mg/dl), elevated inflammatory markers (erythrocyte sedimentation rate [ESR] 103 mm/hr, C-reactive protein [CRP] 5 mg/dl) and normal white blood cell (WBC) count of 6700 cells/uL with elevated absolute eosinophil count of 1820 cells/uL. CT scan of his head was normal.

Despite correction of his hypoglycemia and hypothermia with dextrose infusion and warming blankets for three days, he continued to remain drowsy and minimally responsive. Adrenal insufficiency and bacteremia were ruled out again with an adrenocorticotropic hormone (ACTH) stimulation test and blood cultures. MRI brain and lumbar puncture were performed given his non-resolving encephalopathy and were unremarkable. One persistent finding was a high peripheral blood eosinophil count (up to 31% of his WBC). At this point, HES causing encephalopathy was presumed and empiric steroids were begun. The patient demonstrated marked improvement in his mental status the day after starting steroids by following simple commands and scratching his body. This improvement continued on a daily basis along with a drop in his eosinophil count to 1.5% in 24 hours.

At this point, a workup for the cause of the patient’s hypereosinophilia and the rash was initiated. IgE level was markedly elevated at 3550 (normal <=214 kU/L), stool for ova and parasites and IgG for Strongyloides were negative, and bone marrow biopsy revealed only hypereosinophilia and atypical megakaryocytes. Fluorescence in situ hybridization (FISH) cytogenetics was negative for FIP1L1/PDGFRA fusion (no CHIC2 deletion), and showed normal karyotype and no myelodysplastic features. The very elevated IgE and lack of evidence of clonal disorder on bone marrow biopsy were suggestive of reactive eosinophilia as opposed to a clonal disorder.

**Figure 2:**
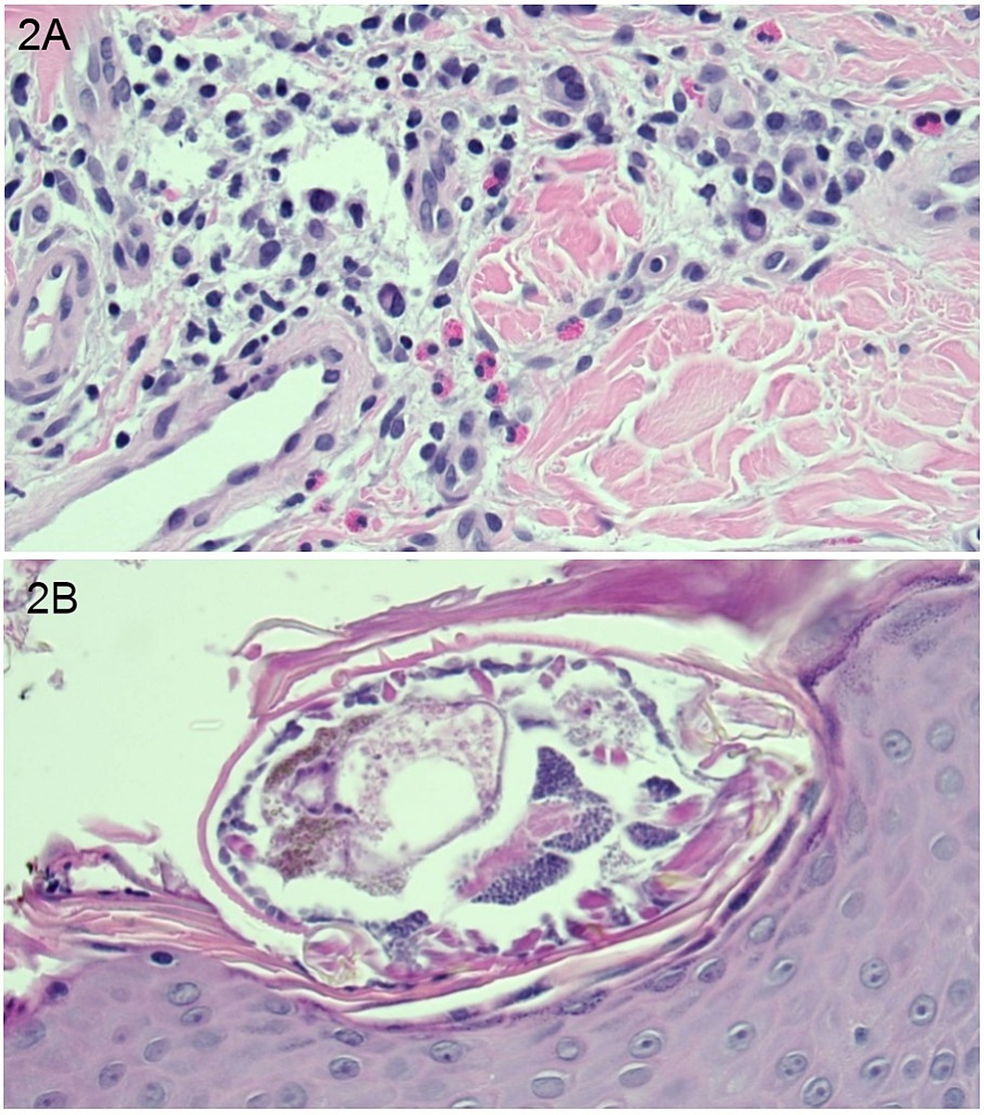
A. Sections from the punch biopsy of skin show acanthosis, spongiosis, hyperkeratosis with intracorneal microabscesses, increased fibrosis of the reticular dermis, and moderate perivascular and dermal inflammation composed of a mix of lymphocytes, plasma cells, and eosinophils. B. Sarcoptes scabiei mites are present within the subcorneal layer.

Skin scrapings were taken from his feet and interdigital spaces to evaluate for scabies and skin punch biopsy from the abdomen for histopathology. The skin scrapings were negative, however, the punch biopsy result a few days later showed fragments of mite consistent with a marked infestation (100 mites/cm2) of Sarcoptes scabiei (Figure 2) without evidence of malignancy. The patient was subsequently placed on contact precautions and begun therapy for crusted scabies.

Our patient presented with a constellation of manifestations including encephalopathy, hypothermia, hypoglycemia, and hypereosinophilia in the setting of a chronic diffuse, pruritic, hyperkeratotic rash consistent with exfoliative erythroderma. The patient’s diffuse exfoliative erythroderma was presumed to be related to his hypereosinophilia and raised the possibilities of cutaneous lymphoma, severe eczema, scabies, or HES-induced erythroderma.

The initial negative metabolic, infectious and malignancy workup for persistent encephalopathy led to the suspicion that his symptoms were likely related to the rather uncommon findings of hypereosinophilia and erythroderma, both present for months to years and up to this point overlooked. Given the negative workup, skin biopsy showing scabies mites, and marked improvement in encephalopathy with steroids a presumptive diagnosis of Scabies induced HES was made.

Treatment of HES is to reduce the level of eosinophils causing end-organ damage by treating the underlying cause of hypereosinophilia. Corticosteroids are the initial choice when patients present with HES. After the first dose of intravenous methylprednisolone, our patient’s mental status and eosinophil count improved markedly. Patients with HES usually respond quickly to corticosteroids with eosinophilia resolving within hours [3]. Our patient was also successfully treated for scabies with topical permethrin and oral ivermectin. He was discharged on oral prednisone 50 mg daily for HES with a planned gradual taper.

**Figure 3:**
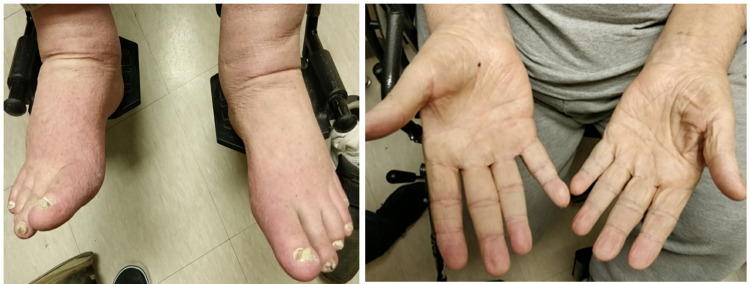
Improvement in the rash during follow-up in clinic in one week.

One week after discharge the patient was seen in the clinic and found to be fully alert, oriented, and conversant with dramatic improvement in his skin lesions. (Figure 3).

## Discussion

HES can present with non-specific variable symptoms such as dermatologic (eczema, erythroderma, lichenification, urticaria), pulmonary (ground-glass infiltrates, fibrosis, pulmonary emboli), gastrointestinal (dysmotility, enteritis, fibrosis), cardiologic (necrotizing myocarditis, mural thrombus, fibrosis, restrictive cardiomyopathy), hematologic (anemia, thrombocytopenia, hepatosplenomegaly) or rheumatologic (vasculitis, joint effusions, Raynaud’s phenomenon) manifestations. Neurological manifestations associated with HES are either cerebral thromboemboli that could arise from intracardiac thrombi; encephalopathy with behavioral changes, confusion, ataxia, and memory loss; or peripheral neuropathy [3]. Our patient had erythroderma, anemia, thrombocytopenia, encephalopathy, hypoglycemia, and hypothermia, which are some of the signs and symptoms of HES.

Workup for HES includes evaluation for secondary causes (e.g., helminth infection, drug hypersensitivity, neoplastic disease) and assessment for end-organ involvement. As no obvious etiology of our patient’s clinical syndrome could be elucidated and initial skin scraping was negative for scabies, we started looking at bone marrow which also did not show any evidence of primary HES. A punch biopsy from an abdominal skin lesion resulted in a few days later showing mites of Sarcoptes scabiei.

Crusted scabies, also known as Norwegian scabies, is a form of scabies with extensive hyperkeratotic skin lesions, crusting, and scaling. Elderly and institutionalized patients who have dementia or are unable to respond to pruritus are prone to develop this form of scabies [4]. The mites in crusted scabies are not more virulent than in classical scabies but are much more numerous (up to 2 million per patient as compared to 10-15 in routine scabies) making crusted scabies highly contagious [5]. Eosinophilia can be seen in over 50% of patients with crusted scabies and serve as a diagnostic clue in the appropriate setting [6]. Although we identified no reports linking scabies to HES, crusted scabies can lead to hypereosinophilia which can be toxic to tissues and lead to systemic illness regardless of the cause.

Diagnosis can be challenging as crusted scabies can resemble other common skin conditions such as drug reactions [7], hyperkeratotic eczema, or plaque psoriasis [8] and, occasionally, as exfoliative erythroderma as observed in our patient. Misdiagnosis, or delayed diagnosis, is common and can lead to serious complications such as hyperinfestation [9], severe secondary infection like Staphylococcus aureus bacteremia [10], and risk of local outbreaks [2]. In our case, prior misdiagnosis was associated with Staphylococcus aureus bacteremia, and the patient was sent to a rehabilitation facility with skin lesions that had a risk of local scabies outbreaks, and the patient also likely developed HES during the course.

## Conclusions

Crusted scabies should be considered in the differential diagnosis of patients presenting with hyperkeratotic, scaly, crusted lesions, especially in institutionalized elderly patients and those with eosinophilia. This disease can be easily overlooked and when missed can lead to complications such as hyperinfestation, bacteremia, local outbreaks, and possibly HES. Eosinophils can be toxic at high levels and lead to HES regardless of the cause, including crusted scabies.

## References

[1] Karimkhani C, Colombara DV, Drucker AM (2017). The global burden of scabies: a cross-sectional analysis from the Global Burden of Disease Study 2015. Lancet Infect Dis.

[2] Cassell JA, Middleton J, Nalabanda A (2018). Scabies outbreaks in ten care homes for elderly people: a prospective study of clinical features, epidemiology, and treatment outcomes. Lancet Infect Dis.

[3] Curtis C, Ogbogu P (2016). Hypereosinophilic syndrome. Clin Rev Allergy Immunol.

[4] Roberts LJ, Huffam SE, Walton SF, Currie BJ (2005). Crusted scabies: clinical and immunological findings in seventy-eight patients and a review of the literature. J Infect.

[5] (2021). Centers for Disease Control and Prevention. Epidemiology and Risk Factors of Scabies. https://www.cdc.gov/parasites/scabies/epi.html.

[6] Seidelman J, Garza RM, Smith CM, Fowler VG Jr (2017). More than a mite contagious: crusted scabies. Am J Med.

[7] Almond DS, Green CJ, Geurin DM, Evans S (2000). Lesson of the week: Norwegian scabies misdiagnosed as an adverse drug reaction. BMJ.

[8] Ebrahim KC, Alves JB, Tomé LA (2016). Norwegian scabies - rare case of atypical manifestation. An Bras Dermatol.

[9] Yari N, Malone CH, Rivas A (2017). Misdiagnosed crusted scabies in an AIDS patient leads to hyperinfestation. Cutis.

[10] Lin S, Farber J, Lado L (2009). A case report of crusted scabies with methicillin-resistant Staphylococcus aureus bacteremia. J Am Geriatr Soc.

